# Exploring Marine-Derived Antioxidant and Anti-Inflammatory Agents: Findings from Recent Studies

**DOI:** 10.3390/md23060240

**Published:** 2025-05-30

**Authors:** Marzia Vasarri, Donatella Degl’Innocenti

**Affiliations:** 1 Department of Experimental and Clinical Biomedical Sciences, University of Florence, Viale Morgagni 50, 50134 Florence, Italy; 2 Interuniversity Center of Marine Biology and Applied Ecology “G. Bacci” (CIBM), Viale N. Sauro 4, 57128 Leghorn, Italy; donatella.deglinnocenti@unifi.it

The Special Issue “Marine Anti-Inflammatory and Antioxidant Agents, 4th Edition” of *Marine Drugs* underscores the immense therapeutic potential embedded within our planet’s vast marine environments. As a prolific reservoir of bioactive compounds, the seas have emerged as a vital source for discovering agents capable of targeting fundamental processes such as inflammation and oxidative stress—key contributors to numerous chronic diseases. This collection of research emphasizes a holistic approach that integrates natural marine compounds, advanced delivery systems, and diverse applications across health domains, illustrating the interconnectedness of immune modulation, antioxidative defense, and disease mitigation.

One of the themes of this Special Issue revolves around marine-derived modulators of immune activation and migration. For instance, in Contribution 1, Liu et al. (2025) demonstrate that alternariol (AOH), a secondary metabolite produced by marine fungi *Alternaria sp.*, can suppress T cell proliferation, cytokine secretion, and migration, thereby reducing lung inflammation in vivo. Such findings highlight the precision with which marine fungal metabolites can modulate immune responses, offering promising strategies for autoimmune and allergic conditions. Complementing this, Niu et al. (2024) in Contribution 2 investigate Butyrolactone-I (BTL-I), a bioactive compound from deep-sea fungus *Aspergillus* C23-3, which protects intestinal cells and mice from heat-stress-induced oxidative damage and apoptosis. This work reveals that BTL-I reduces heat stress markers (HSP70 and HSP90), diminishes oxidative stress indicators (ROS and MDA), and enhances antioxidant enzyme activity (SOD). Importantly, BTL-I attenuates apoptosis, thereby bolstering intestinal resilience under stress—an approach aligned with the sustainable management of livestock in the context of climate change.

The scope of marine bioactivities extends into natural antioxidants that can be used to promote skin health and provide systemic protection. In Contribution 3, Yang et al. (2024) provide compelling evidence that a peptide derived from seahorse (*Hippocampus abdominalis*) hydrolysate (SHP2) mitigates UVB-induced skin damage by reducing ROS levels, improving cell viability, and promoting collagen synthesis in human keratinocytes and fibroblasts. This work also demonstrates photoaging inhibition in zebrafish models, highlighting SHP2’s potential in cosmetic formulations aimed at preventing skin photoaging. Furthermore, marine-derived peptides from the blue mussel Mytilus edulis are shown to exhibit promising cardiovascular benefits in Contribution 4 by Marasinghe et al., 2024; they inhibit foam cell formation and inflammation in macrophages by modulating lipid metabolism and inflammatory pathways, showcasing the translational potential of marine biochemistry in cardiovascular health.

Nutritional applications of marine resources also feature prominently. In Contribution 5, Joshi et al. (2025) develop an enzymatically stabilized squid oil enriched with omega-3 fatty acids and astaxanthin, which demonstrates enhanced oxidative stability and nutritional retention, supporting dietary strategies to combat inflammation.

Similarly, in Contribution 6, Shu et al. (2024) explore a sea cucumber polydeoxyribonucleotide from *Apostichopus japonicus* sperm (AJS-PDRN), revealing its potent free radical scavenging properties and capacity to protect macrophages from oxidative damage. Their findings suggest that AJS-PDRN can modulate immune responses, promote membrane repair, and bolster antioxidant defenses through multiple pathways.

Further exploring anti-inflammatory and antioxidant agents, in Contribution 7, Dörschmann et al. (2025) investigate a very high-molecular-weight fucoidan (FucBB04) from the brown seaweed *Laminaria hyperborea* for potential therapeutic effects in age-related macular degeneration (AMD) models in vitro. While FucBB04 reduced inflammatory cytokine secretion (IL-6 and IL-8) and modulated VEGF levels, it did not provide antioxidant protection and negatively impacted key retinal pigment epithelium (RPE) functions. These findings suggest that although high-molecular-weight fucoidans exhibit some bioactivity against AMD-related pathways, their adverse effects on RPE cell functions highlight the need for further research with smaller-molecular-weight variants. Complementary to these findings, alginate extracted from the brown seaweed *Ericaria crinita* by Lukova et al. (2024) in Contribution 8 shows promise due to its free radical scavenging and cytokine-modulating activities, notably reducing pro-inflammatory cytokines like TNF-α and IL-6 in models of systemic inflammation. These insights point toward marine polysaccharides as versatile candidates for anti-inflammatory therapies, although further mechanistic studies are necessary.

Among research on seaweeds bioactivities, Lee et al. (2025) demonstrate in Contribution 9 that a water extract of the green seaweed *Codium fragile* (WCF), rich in bioactive oleamide (9-octadecenamide), exhibits significant anti-inflammatory, antioxidant, and anti-fibrotic effects in a mouse model of allergic asthma induced by ovalbumin. WCF modulates immune responses by reducing Th2 cytokines, eosinophil infiltration, and IgE levels, while alleviating airway inflammation, mucus overproduction, and tissue fibrosis. Mechanistically, it suppresses the TLR4/NF-κB and TGF-β1/Smad signaling pathways, decreases pro-inflammatory cytokines, oxidative stress markers, and fibrosis-related proteins, and protects against apoptosis. Overall, WCF shows promise as a natural therapeutic agent for allergic respiratory diseases.

Expanding on anti-inflammatory strategies, in Contribution 10, Pruvost et al. (2024) illustrate that nanocarriers composed of chondroitin sulfate and lipids derived from salmon (*Salmo salar*) can significantly diminish inflammatory markers in human chondrocytes. This research exemplifies how marine polysaccharides and lipids synergistically attenuate joint inflammation, reinforcing the therapeutic promise of marine biochemicals.

The fourth edition of “Marine Antioxidant and Anti-Inflammatory Agents” highlights a wide variety of marine organisms that serve as sources of bioactive compounds with antioxidant and anti-inflammatory properties. The broad diversity of species and molecules listed in Table 1 emphasizes the valuable resource that the marine environment represents in the field of human health.

To harness the vast potential of marine bioresources in the search for new anti-inflammatory and antioxidant agents, we are pleased to present two in-depth reviews included in this Special Issue.

In order to achieve this purpose, Yang et al. (2024) present in Contribution 11 a comprehensive review of marine microorganisms, thereby demonstrating that more than 250 microbial metabolites—including polyketides, terpenoids, alkaloids, amides or peptides, and steroids—have shown promising pharmacological activities, thus enriching the pipeline for future drug development.

Furthermore, in Contribution 12, Vasarri et al. (2025) conduct a study that emphasizes the sustainable availability of seagrasses as natural sources of bioactive compounds. This review is an investigation into the antioxidant and anti-inflammatory properties of Mediterranean seagrasses, including *Posidonia oceanica*, *Cymodocea nodosa*, and *Zostera* species. It is demonstrated that the utilization of these seagrasses holds considerable potential for the mitigation of oxidative stress and chronic inflammation.

Collectively, these reviews emphasize the substantial and as yet underutilized capacity of marine ecosystems in the production of anti-inflammatory and antioxidant agents.

In conclusion, the diverse array of marine-derived bioactivities highlighted in this Special Issue underscores the profound therapeutic potential residing within marine environments. From fungal metabolites and marine peptides to polysaccharides and microbially produced compounds, these several natural products from diverse marine organisms (Figure 1) offer novel mechanisms to combat inflammation and oxidative stress—central contributors to many chronic diseases. The integration of advanced delivery systems, sustainable sourcing, and multidisciplinary research approaches further enhances their translational prospects. As our understanding of marine biochemistry deepens, the utilization of these bioresources has the potential to facilitate innovative and efficacious applications in human health, while concurrently contributing to marine ecological preservation.

## Figures and Tables

**Figure 1:**
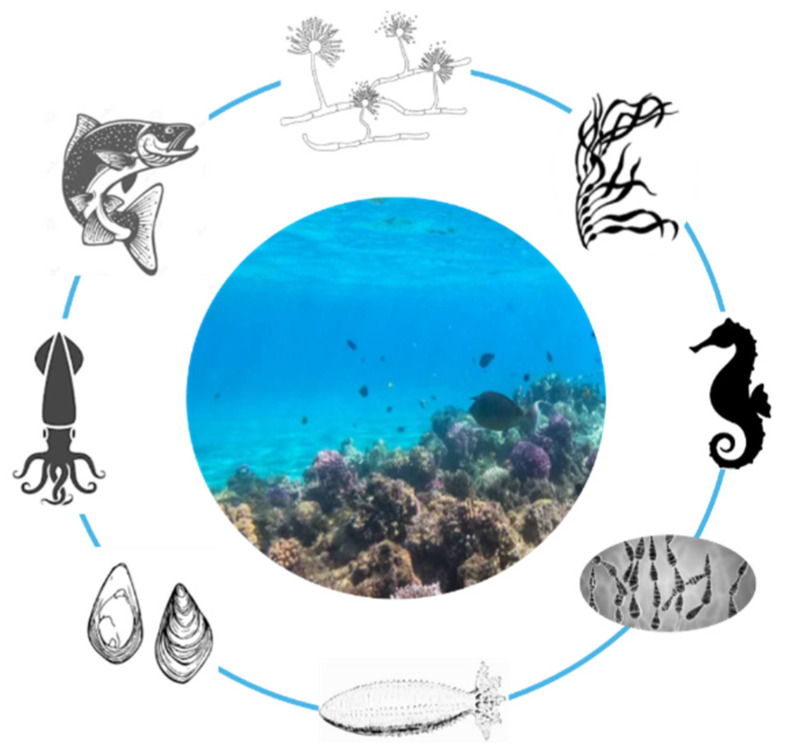
Scheme illustrating of the key marine organisms as sources of bioactive compounds with antioxidant and anti-inflammatory properties featured in the Special Issue “Marine Anti-Inflammatory and Antioxidant Agents, 4th Edition”.

**Table 1:** Summary of the research collected in the Special Issue “Marine Anti-Inflammatory and Antioxidant Agents, 4th Edition”.

Marine Organism	Compound	In Vitro Experimental Model	In Vivo Animal Models	Contribution
Marine fungi *Alternaria* sp.	Alternariol (AOH)	Bone marrow-derived dendritic cells (BMDCs) and macrophages (BMDMs)	C57BL/6J mice	Contribution 1 (Liu et al. (2025))
Marine fungus Aspergillus C23-3	Butyrolactone-I (BTL-I)	Intestinal porcine enterocyte (IPEC-J2) cells	C57BL/6J mice	Contribution 2 (Niu et al. (2024))
Seahorse (*Hippocampus abdominalis*)	SHP2 peptide	Human keratinocyte (HaCaT) cells; Human dermal fibroblast (HDF) cells	Zebrafish model	Contribution 3 (Yang et al. (2024))
Blue mussel (*Mytilus edulis*)	Peptides PIISVYWK (P1) and FSVVPSPK (P2)	Murine macrophage (RAW264.7) cells		Contribution 4 (Marasinghe et al. (2024))
Squid viscera	oil enriched with omega-3 fatty acids and astaxanthin			Contribution 5 (Joshi et al. (2025))
Sea Cucumber (*Apostichopus japonicus*)	Polydeoxyribonucleotide (AJS-PDRN)	Murine macrophage (RAW264.7) cells		Contribution 6 (Shu et al. (2024))
Brow seaweed (*Laminaria hyperborea)*	Fucoidan (FucBB04)	Human uveal melanoma (OMM-1) cells; primary retinal pigment epithelium (RPE) cells from pig eyes; immortalized retinal pigment epithelial (ARPE-19) cells		Contribution 7 (Dörschmann et al. (2025))
Brown seaweed (*Ericaria crinita)*	Alginate		Wistar rats	Contribution 8 (Lukova et al. (2024))
Green seaweed (*Codium fragile*)	oleamide (9-octadecenamide)		BALB/c mice	Contribution 9 (Lee et al. (2025))
Salmon (*Salmo salar*)	chondroitin sulfate and lipids	Primary human chondrocyte cells		Contribution 10 (Pruvost et al. (2024))

